# Supplementary material for: Progression of Pulmonary Function and Correlation with Survival Following Stereotactic Body Radiotherapy of Central and Ultracentral Lung Tumors
Source: Cancers (Basel). 2020 Oct 5;12(10):2862. doi: 10.3390/cancers12102862 (PMC7600477; doi:10.3390/cancers12102862)
Supplement: Supplementary file 1 [file cancers-12-02862-s001.pdf]

cancers

MDPI

Progression of Pulmonary Function and Correlation with Survival Following Stereotactic Body Radiotherapy of Central and Ultracentral Lung Tumors

Sebastian Regnery, Tanja Eichkorn, Fabian Weykamp, Thomas Held, Lisa-Antonia Dinges, Fabian Schunn, Hauke Winter, Michael Thomas, Jürgen Debus, Rami A. El Shafie, Sebastian Adeberg and Juliane Hörner-Rieber

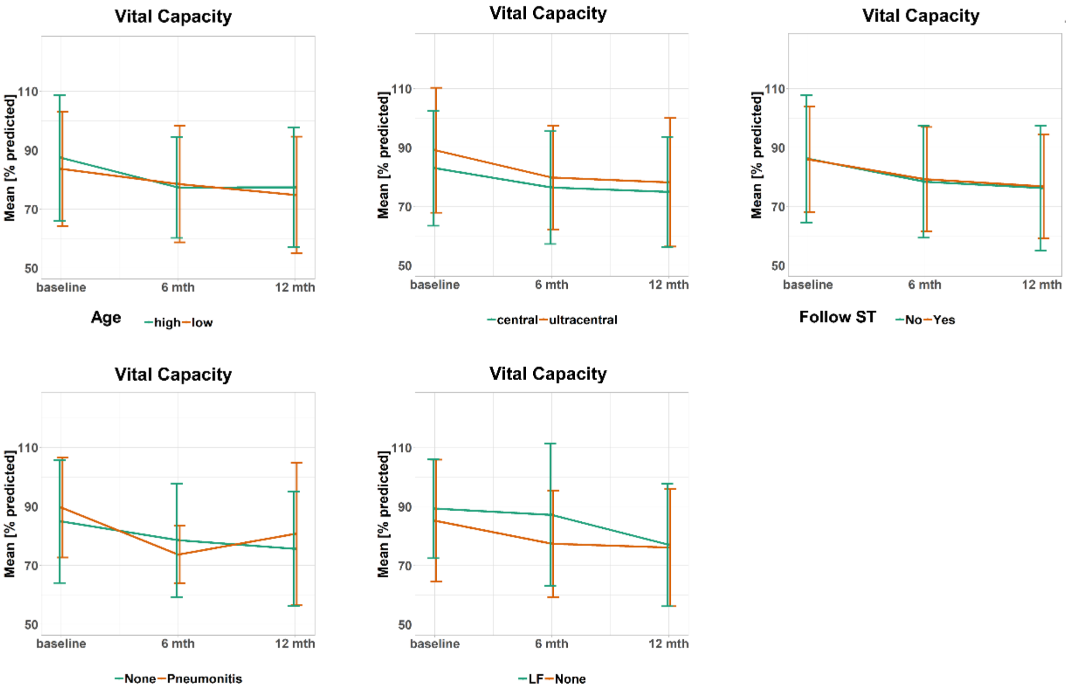

Figure S1. Progression of vital capacity for further patient subgroups. Data points represent the mean value at a given time point. Error bars visualize ± 1 standard deviation. Patient age was dichotomized at the median to obtain one group with higher and one with lower age. Follow ST: systemic therapy during the PFT follow-up. LF: local failure.

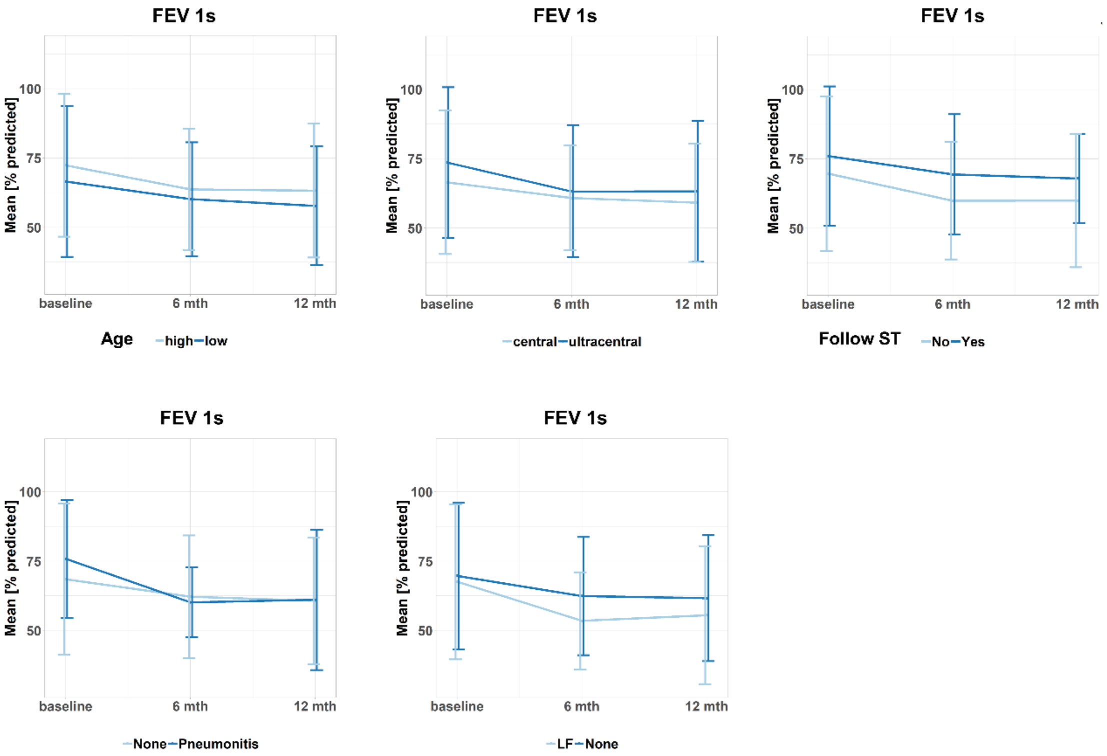

Figure S2. Progression of forced expiratory volume in the first second (FEV 1s) for further patient subgroups. Data points represent the mean value at a given time point. Error bars visualize ± 1 standard deviation. Patient age was dichotomized at the median to obtain one group with higher and one with lower age. Follow ST: systemic therapy during the PFT follow-up period. LF: local failure.

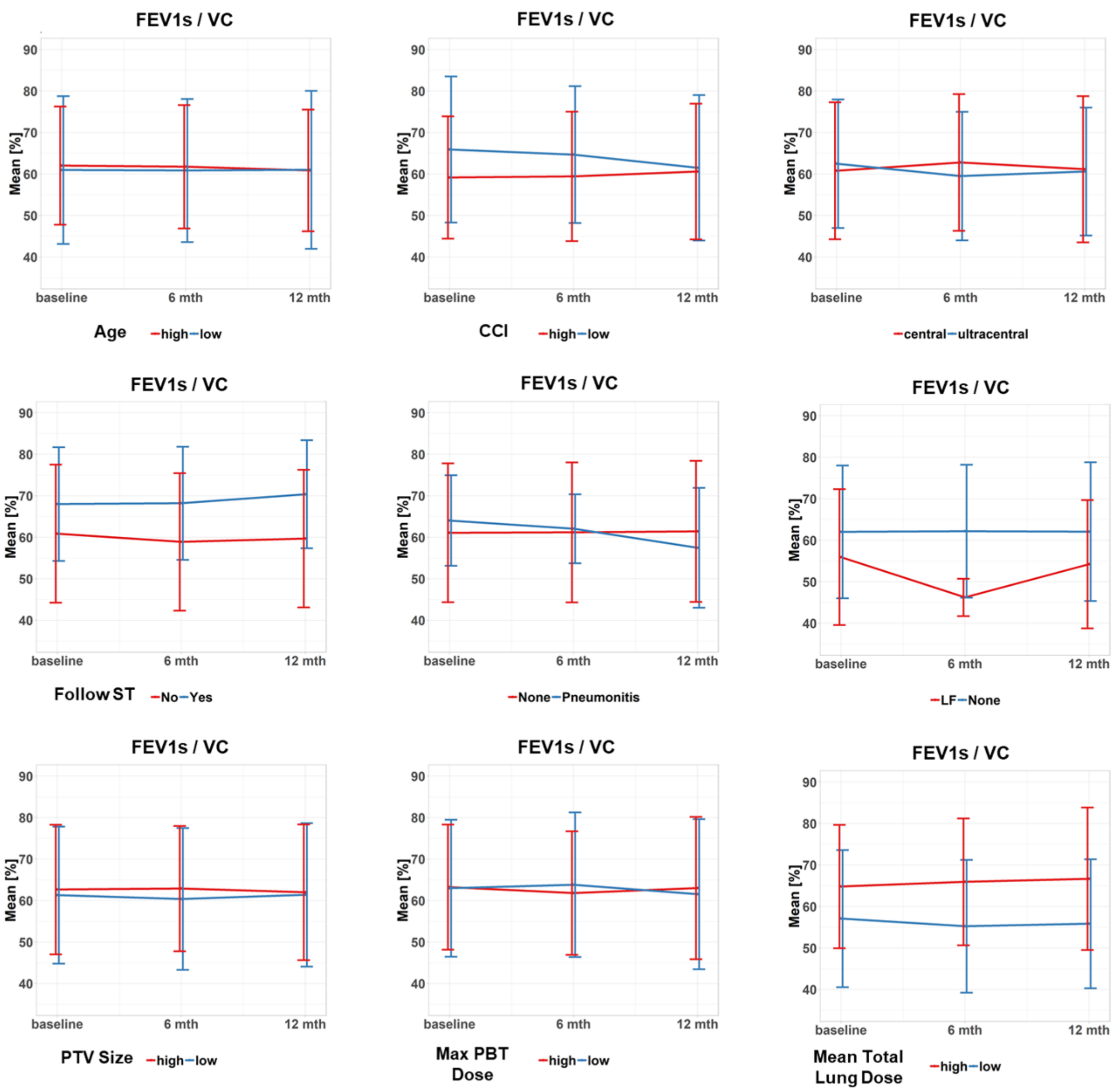

Figure 3. Progression of the forced expiratory volume in the first second to vital capacity ratio (FEV1s/VC) for different subgroups. Data points represent the mean value at a given time point. Error bars visualize ± 1 standard deviation. The continuous variables were dichotomized at the median to obtain two groups, one with high and one with low values of the variable. CCI: Charlson Comorbidity Index, Follow ST: systemic therapy during the PFT follow-up period. LF: local failure. PTV: planning target volume. PBT: proximal bronchial tree.

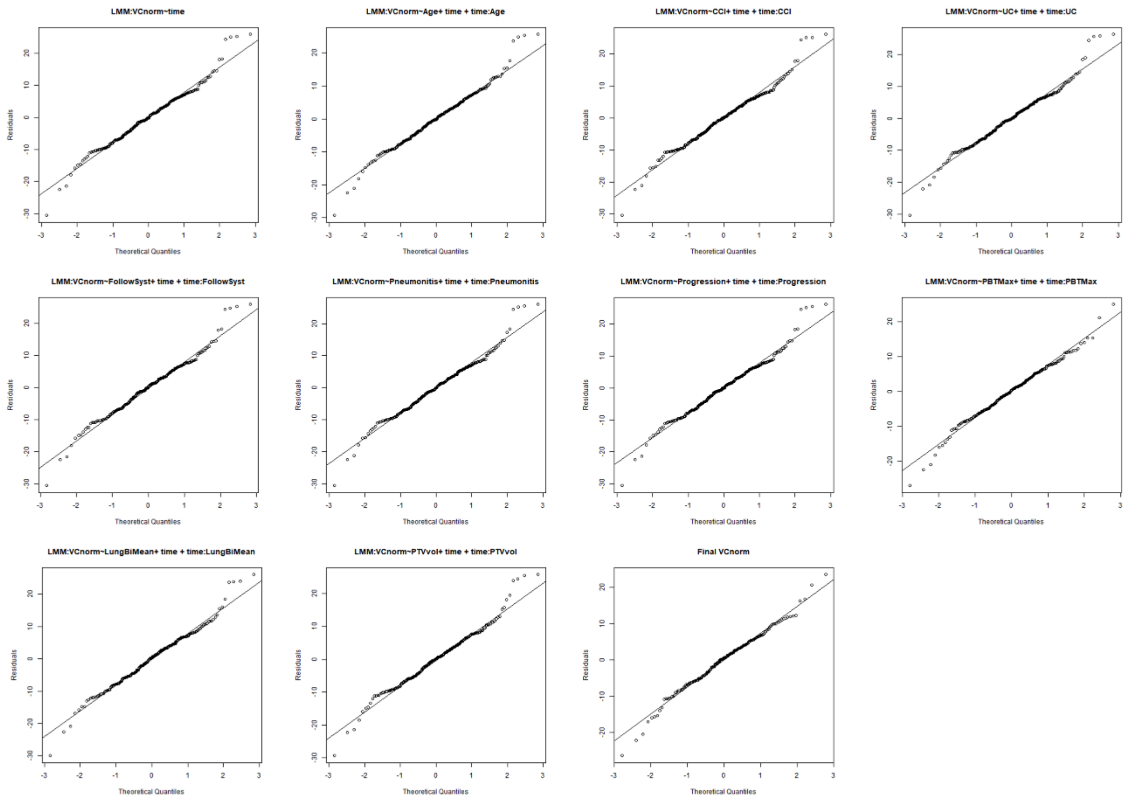

Figure 4. Q-Q-Plots of residuals for the linear mixed models (LMM) of the normalized vital capacity (VCnorm). CCI: Charlson Comorbidity Score, UC: ultracentral localization, FollowSyst: systemic therapy during PFT follow-up, PBTMax: maximum BED_3 to proximal bronchial tree, LungBiMean: Mean BED_3 in total lung, PTVvol: size of planning target volume.

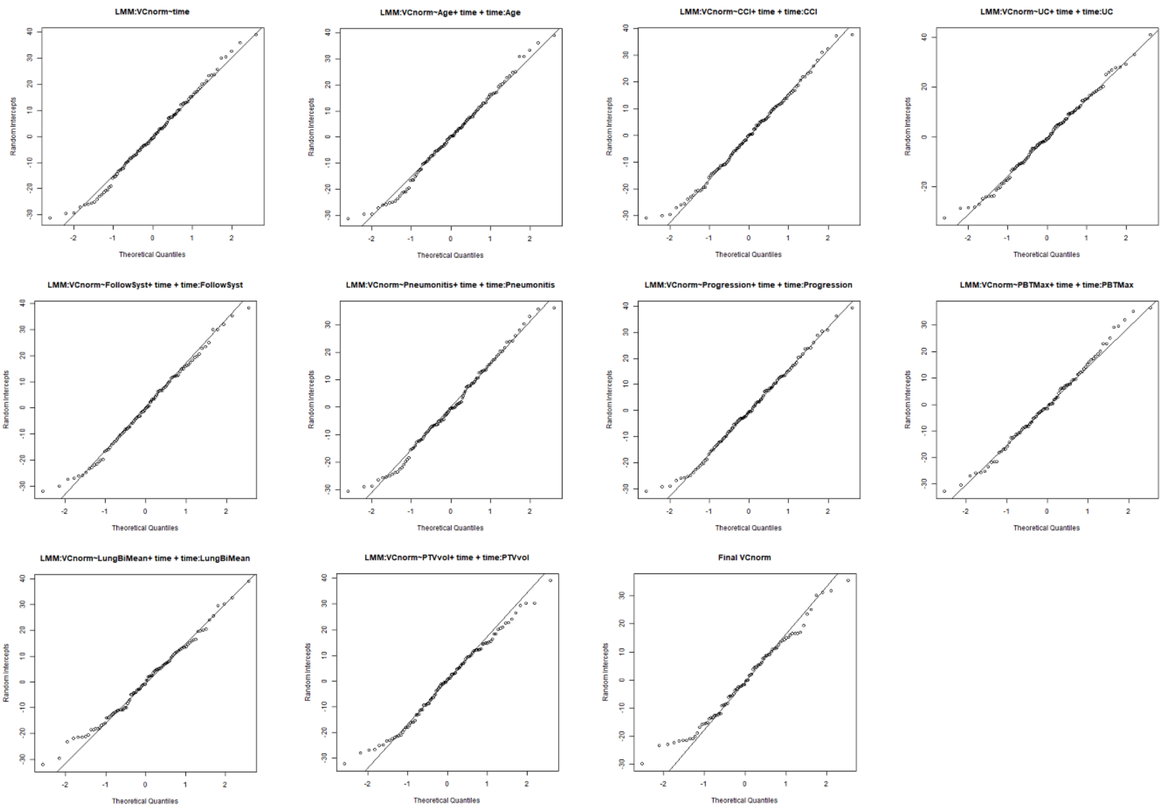

Figure S5. Q-Q-Plots of random intercepts for the linear mixed models (LMM) of the normalized vital capacity (VCnorm). CCI: Charlson Comorbidity Score, UC: ultracentral localization, FollowSyst: systemic therapy during PFT follow-up, PBTMax: maximum BED_3 to proximal bronchial tree, LungBiMean: Mean BED_3 in total lung, PTVvol: size of planning target volume.

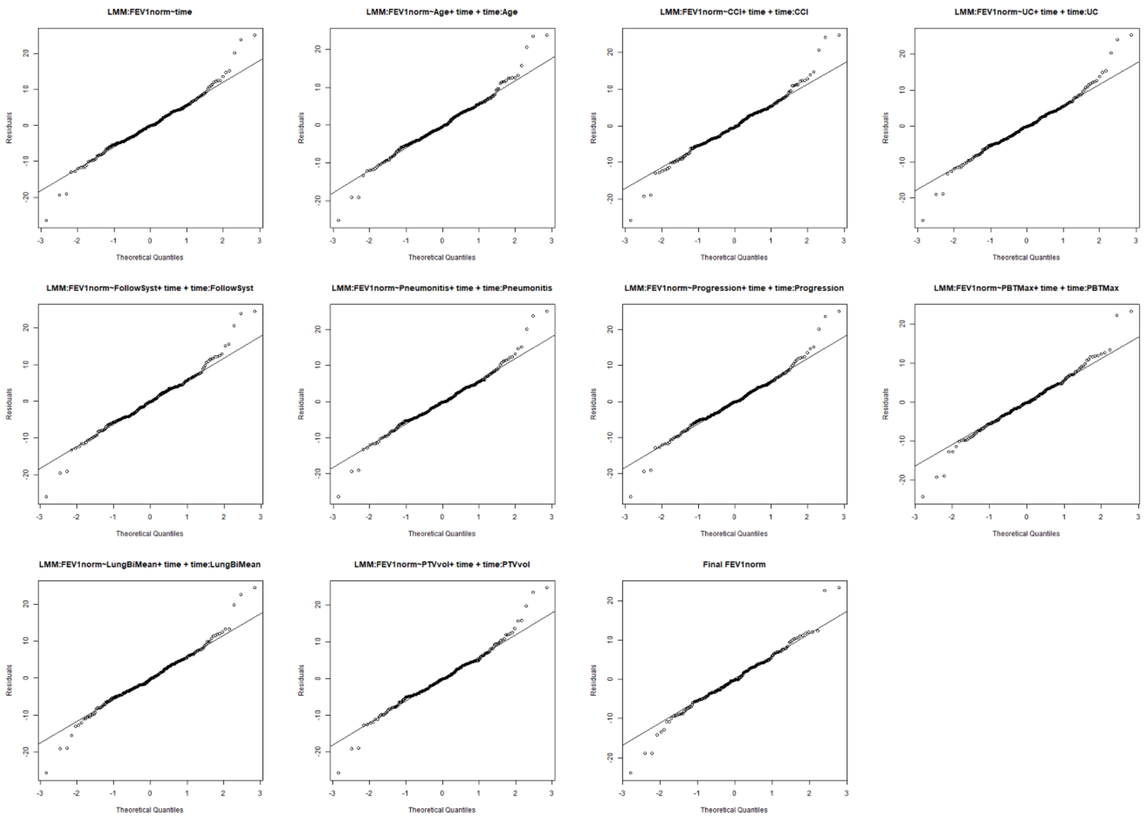

Figure S6. Q-Q-Plots of residuals for the linear mixed models (LMM) of the normalized forced expiratory volume in 1 second (FEV1norm). CCI: Charlson Comorbidity Score, UC: ultracentral localization, FollowSyst: systemic therapy during PFT follow-up, PBTMax: maximum BED_3 to proximal bronchial tree, LungBiMean: Mean BED_3 in total lung, PTVvol: size of planning target volume.

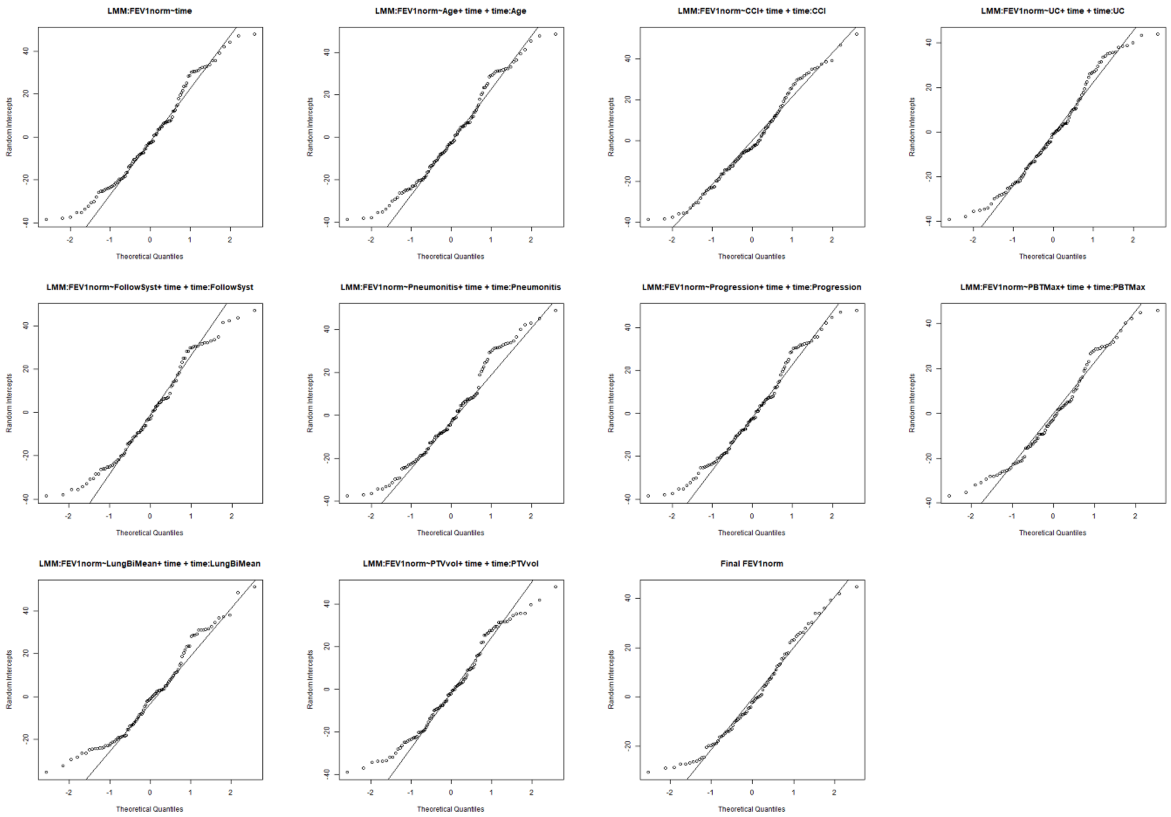

Figure S7. Q-Q-Plots of random intercepts for the linear mixed models (LMM) of the normalized forced expiratory volume in 1 second (FEV1norm). CCI: Charlson Comorbidity Score, UC: ultracentral localization, FollowSyst: systemic therapy during PFT follow-up, PBTMax: maximum BED_3 to proximal bronchial tree, LungBiMean: Mean BED_3 in total lung, PTVvol: size of planning target volume.

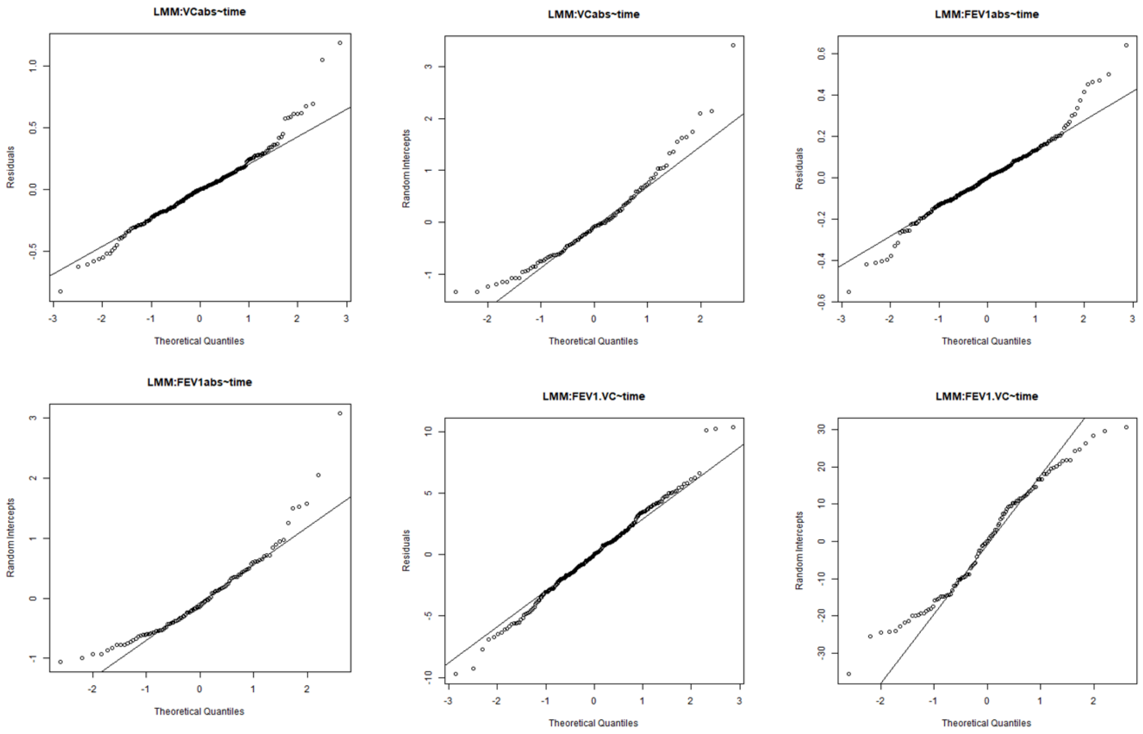

Figure 8. Q-Q-Plots of random intercepts and residuals for the linear mixed models (LMM) incorporating only time after SBRT. FEV1abs: Absolute forced expiratory volume in 1 second, VCabs: absolute vital capacity, FEV1s.VC: ratio between forced expiratory volume in 1 second and vital capacity.

© 2020 by the authors. Licensee MDPI, Basel, Switzerland. This article is an open access article distributed under the terms and conditions of the Creative Commons Attribution (CC BY) license (http://creativecommons.org/licenses/by/4.0/).
